# Longitudinal effects of obesity on pulmonary function in obese children and adolescents

**DOI:** 10.1038/s41390-024-03544-2

**Published:** 2024-09-07

**Authors:** Pawinee Charoensittisup, Kanokporn Udomittipong, Khunphon Mahoran, Apinya Palamit

**Affiliations:** Division of Pulmonology, Department of Pediatrics, Faculty of Medicine Siriraj Hospital, Mahidol University, Bangkok, Thailand

## Abstract

**Background:**

To investigate the longitudinal effects of obesity on change in lung function after 1 year of follow-up in obese children and adolescents.

**Methods:**

Obese children/adolescents aged 8–15 years with pulmonary function test (PFT) results and recorded anthropometric obesity indices from 1 year earlier for comparison were recruited. Multiple linear regression of change in each lung function parameter was applied to determine the effect of sex, change in body mass index (ΔBMI), change in chest circumference (ΔCC), change in waist circumference (ΔWC), and change in waist circumference-to-height ratio (ΔWC/Ht).

**Results:**

Sixty-six children/adolescents (mean age: 12.5 ± 2.6 years) were recruited. Multiple linear regression analysis showed that ΔWC negatively affects the ratio of the forced expiratory volume in the first 1 s to the forced vital capacity of the lungs Δ(FEV_1_/FVC) (*b* = −0.3, *p* = 0.002), forced expiratory flow rate within 25–75% of vital capacity (ΔFEF_25–75%_) (*b* = −0.92, *p* = 0.006), and Δ(FEF_25–75%_/FVC) (*b* = −0.99, *p* = 0.003). When replacing ΔWC with Δ(WC/Ht) as the independent variable, Δ(WC/Ht) also negatively affects Δ(FEV_1_/FVC) (*b* = −33.71, *p* = 0.02), ΔFEF_25–75%_ (*b* = −102.9, *p* = 0.03) and Δ(FEF_25–75%_/FVC) (*b* = −102.7, *p* = 0.03).

**Conclusion:**

After 1 year of follow-up, change in abdominal adiposity determined by WC and WC/Ht exerted significant negative effect on lung function change specific to FEV_1_/FVC, FEF_25–75%_ /FVC, and FEF_25–75%_.

**Impact:**

- Longitudinal effects of change in obesity on lung function in obese children and adolescents are evidenced.
- Change in waist circumference or waist-to-height ratio, which indicates abdominal adiposity, was inversely correlated with a change in FEV1/FVC, FEF25–75% /FVC, and FEF25–75% in children and adolescents with obesity after 1 year of follow-up.
- Our results suggest using waist circumference and/or waist-to-height ratio in addition to BW and/or BMI for monitoring obesity.
- Fat loss programs, especially those focused on reducing abdominal adiposity should be encouraged to prevent late lung function impairment.

## Introduction

Obesity is a major global public health problem for children, adolescents, and adults, and its prevalence continues to increase. In addition to the functional impact on activities of daily living, obesity can cause significant health problems to various systems of the body.^1^ One of the most important systems to be adversely affected by obesity is the respiratory system with commonly observed problems that include obstructive sleep apnea, exercise intolerance, and altered lung function.^2,3^

Impaired lung function can also be observed in obese children and adolescents, including reductions in forced expiratory volume in 1 s/forced vital capacity (FEV_1_/FVC) ratio, functional residual capacity, expiratory reserve volume (ERV), and residual volume (RV).^4,5^ Most previous studies in the effects of obesity on lung function in children and adolescents had a cross-sectional design, and the results were often inconclusive. The long-term effects of obesity on changes in lung function were mostly studied in adults following bariatric surgery,^6^ which is a surgical procedure that is less commonly performed in children and adolescents. A longitudinal study in children by van de Griendt, et al.^7^ found a negative correlation between ERV change and both the change in waist circumference (WC) and the standard deviation score-body mass index (SDS-BMI), but no correlation with spirometric parameters. The Dutch Prevention and Incidence of Asthma and Mite Allergy (PIAMA)^8^ and Swedish BAMSE (Swedish abbreviation for Children, Allergy, Milieu, Stockholm, Epidemiology)^9^ studies found persistent obesity or high body mass index (BMI) to be associated with a lower forced expiratory volume in 1 s/forced vital capacity (FEV_1_/FVC) ratio.

How long-term change in weight and body composition is associated with lung function change in obese children and adolescents remains unclear, and the currently available published data on this important topic remains scarce. Accordingly, the aim of this study was to investigate the longitudinal effects of change in obesity on lung function parameters, including spirometry, respiratory muscle strength (RMS), and 6-min walk test (6-MWT), after a 1-year follow-up in obese children and adolescents. The results of this study will yield important data that will help us develop and improve strategies for managing children and adolescents with obesity.

## Material and methods

### Study protocol

This study was conducted at the Division of Pulmonology of the Department of Pediatrics, Faculty of Medicine Siriraj Hospital, Mahidol University, Bangkok, Thailand during January 2023 to January 2024. Inform consent or assent was obtained from participants and/or their legal guardian(s) before study enrollment. The study protocol was reviewed and approved by the Siriraj Institutional Review Board (approval no. Si 878/2022).

This follow-up study recruited non-syndromic obese children and adolescents aged 8–15 years who had acceptable and reproducible pulmonary function test (PFT) and recorded anthropometric obesity indices 1 year (±3 months) earlier for comparison. For this study, PFT and obesity indices were remeasured on the appointed study date so that the new measurement data could be compared with the measurement data collected one year prior. Obesity was defined as BMI z-score ≥2 according to World Health Organization (WHO) criteria.^10^ We excluded patients with a history of cardiac, neuromuscular, or pulmonary diseases; respiratory infections within the preceding 4 weeks; history of active or passive smoke; and/or, inability or unwillingness to perform the proposed study tests. All enrolled study patients were given standard treatment for obesity, including nutritional consultation for dietary control, recommendation for exercise and lifestyle modification, and evaluation of comorbidities. They may receive treatment for other specific diseases such as positive airway pressure for obstructive sleep apnea, intranasal steroid/ antihistamine for allergic rhinitis, or antihypertensive medications for hypertension.

Collected data included age, sex, height, and obesity indices, including body weight (BW), BMI, BMI z-score, chest circumference (CC), WC, and waist-to-height ratio (WC/Ht). PFTs, including spirometry, 6-MWT, and RMS, were performed by the same technician during the follow-up remeasurement period (i.e., the current study).

### Anthropometric evaluation

**Fig. 1:**
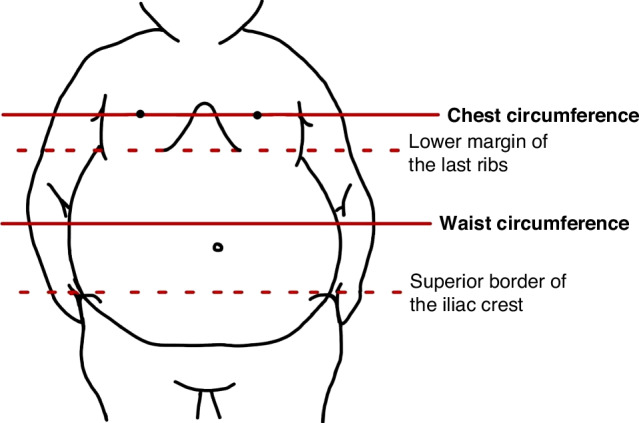
Body level for measuring chest circumference and waist circumference. The figure depicts the body level for measuring chest circumference and waist circumference. Chest circumference is measured at nipple level. Waist circumference is measured between the lower margin of the last ribs and the superior border of iliac crest.

Standardized measurement tools (TANITA Corporation, Tokyo, Japan) were used to measure BW and height. BMI was calculated as BW (kg) divided by the square of height (m^2^). BMI was also expressed as z-score (BMI z-score), which is the BMI adjusted for age and sex according to WHO growth reference data.^11^ CC was measured at nipple level, and WC was measured between the lower margin of the last rib and the iliac crest (Fig. 1)– both measured using a tape measure.

### Spirometry

A Vyntus^TM^ BODY Plethysmograph (Vyaire Medical, Mettawa, IL) was used for spirometry according to American Thoracic Society (ATS)/European Respiratory Society (ERS) recommendations.^12^ The collected spirometry data included forced expiratory volume in 1 s (FEV_1_), forced vital capacity (FVC), FEV_1_/FVC ratio, forced expiratory flow rate within 25–75% of vital capacity (FEF_25–75%_). Except for FEV_1_/FVC ratio, all parameters were reported as percentage of predicted value (%predicted) calculated from multiethnic global lung function 2012 equations.^13^

### 6-min walk test (6-MWT)

The 6-MWT, which is a recommended performance-based tool for functional fitness assessment in accordance with the ATS guideline, was performed.^14^ Participants were instructed to walk as fast as possible without running for 6 min on a flat and straight surface. The length of the walking course was 30 meters with a clearly visible cone placed at the end of the course. During the test, participants were encouraged to walk, and the time remaining was announced at different timepoints. This test was immediately stopped if participants had shortness of breath, chest pain, heart palpitations, or leg cramps. The total distance walked was measured after 6 min of walking. The 6-MWT distance were reported as absolute and z-score value according to reference equation.^15^

### Respiratory muscle strength (RMS)

Inspiratory and expiratory muscle strength was assessed via maximal inspiratory pressure (MIP) and maximal expiratory pressure (MEP), respectively, using a Vyntus^TM^ BODY Plethysmograph (Vyaire Medical) according to ATS/ERS recommendations.^16^ MIP was measured with the subject breathing in from RV to total lung capacity (TLC), and MEP was measured during forced expiration from TLC to RV. The measurement considered for data analysis was the highest value among 3 acceptable maneuvers (without leakage and lasting for at least 1 s), and at least two were reproducible (not different more than 10% from the second highest value). MIP and MEP values were expressed as absolute value and %predicted value based on reference equation.^17^

### Statistical analysis

The data are presented as mean plus/minus (±) standard deviation (SD) for normally distributed continuous data, and as number and percentage for categorical data. Student’s *t*-test was used to compare baseline and follow-up data. Multiple linear regression was used to identify the effects of the independent variables, including sex, change in body mass index (ΔBMI), change in chest circumference (ΔCC), change in waist circumference (ΔWC), and change in waist circumference-to-height ratio (ΔWC/Ht), on each lung function parameter. Those results are shown as correlation coefficient (b) and *p*-value. Multicollinearity between independent variables was checked using variance inflation factor. SPSS Statistics for Windows version 26.0 (SPSS, Inc., Chicago, IL) was used to analyze the data. A *p* < 0.05 was considered statistically significant for all tests.

## Results

### Study group

This study enrolled 66 obese children and adolescents (50 boys, and 16 girls) with a mean age of 12.5 ± 2.6 years at follow-up. BMI was reduced in 35 participants, and increased in 31 participants with the mean change of 1.78 ± 1.28 kg/m^2^ and 1.28 ± 1.10 kg/m^2^ respectively, after a mean follow-up duration of 1.01 ± 0.14 years. Obesity indices that were statistically significantly different from baseline to follow-up included bodyweight (77.4 vs. 80.5 kg, *p* < 0.001), BMI z-score (3.56 vs. 3.20 kg/m^2^, *p* < 0.001), CC (98.1 vs. 99.6 cm, *p* = 0.049), and WC/Ht (0.66 vs. 0.65, *p* = 0.029) (Table 1).

**Table 1:** Demographic characteristics and anthropometric obesity indices compared between baseline and the 1-year follow-up (*N* = 66).

Characteristics and obesity indices	Mean ± standard deviation		*p*-value
	Baseline	1-year follow-up	
Age (years)	11.5 ± 2.5	12.5 ± 2.6	-
Height (meters)	1.53 ± 0.15	1.58 ± 0.14	-
Weight (kg)	77.4 ± 26.0	80.5 ± 23.9	**<0.001**
Body mass index (kg/m^2^)	31.9 ± 6.2	31.6 ± 5.7	0.15
Body mass index z-score	3.56 ± 1.08	3.20 ± 0.96	**<0.001**
Chest circumference (cm)	98.1 ± 14.3	99.6 ± 14.4	**0.049**
Waist circumference (cm)	102.1 ± 15.0	102.9 ± 14.4	0.32
Waist circumference/height	0.66 ± 0.07	0.65 ± 0.07	**0.03**

A *p* < 0.05 indicates statistical significance. The bold means a *p* < 0.05.

A comparison of lung function between baseline and the follow-up showed a significant increase in MIP (106.2 vs. 118.1 cmH_2_O, *p* < 0.001, and 115.7 vs. 126.8%predicted, *p* < 0.001), MEP (100.6 vs. 110.0 cmH_2_O, *p* < 0.001, and 100.0 vs. 108.4%predicted, *p* < 0.001), and 6-MWT distance (533.9 vs. 554.2 meters, *p* = 0.004). The spirometry parameters that were significantly decreased at follow-up included FEV_1_/FVC (84.1 vs. 82.5%, *p* = 0.008) and FEF_25–75%_/FVC (92.7 vs. 87.2%, *p* = 0.007) (Table 2).

**Table 2:** Pulmonary function test parameters compared between baseline and the 1-year follow-up (*N* = 66).

Pulmonary function parameters	Mean ± standard deviation		*p*-value
	Baseline	1-year follow-up	
MIP (cmH_2_O)	106.3 ± 30.5	118.1 ± 29.9	**<0.001**
MIP (% predicted)	115.7 ± 31.0	126.8 ± 28.7	**<0.001**
MEP (cmH_2_O)	100.6 ± 28.8	110.0 ± 30.2	**<0.001**
MEP (% predicted)	100.0 ± 24.2	108.4 ± 23.1	**<0.001**
FVC (% predicted)	111.3 ± 13.4	113.1 ± 11.6	0.07
FEV_1_ (% predicted)	105.1 ± 14.1	104.8 ± 12.9	0.74
FEV_1_/FVC (%)	84.1 ± 7.2	82.5 ± 7.0	**0.008**
FEF_25–75%_ (% predicted)	91.3 ± 23.3	88.3 ± 23.3	0.14
FEF_25–75%_ /FVC (%)	92.7 ± 25.1	87.2 ± 24.1	**0.007**
PEF (% predicted)	87.4 ± 26.3	83.7 ± 19.2	0.16
6-MWT distance (meters)	533.9 ± 50.9	554.2 ± 54.0	**0.004**
6-MWT z-score	0.2 ± 0.8	0.4 ± 0.8	0.091

A *p* < 0.05 indicates statistical significance. The bold means a *p* < 0.05.

*6-MWT* distance 6-min walk test distance, *FEV*_*1*_ forced expiratory volume in 1 s, *FEF*_*25–75%*_ forced expiratory flow rate within 25–75% of vital capacity, *FVC* forced vital capacity, *MEP* maximal expiratory pressure, *MIP* maximal inspiratory pressure, *PEF* peak expiratory flow rate.

No participants received weight loss pharmacotherapy or underwent surgery for the treatment of obesity.

### Factors related to change in PFTs

Table 3 shows the results of multiple linear regression analysis of changes in lung function on two different sets of independent variables. Model 1 included sex, ΔBMI, ΔCC, and ΔWC. Model 2 included sex, ΔBMI, ΔCC, and Δ(WC/Ht).

**Table 3:** Multiple linear regression analysis to identify the anthropometric obesity variables that independently associate with significant change in pulmonary function parameters.

Δ in pulmonary function parameters	Independent variables								
	Sex: M		ΔBMI (kg/m^2^)			ΔCC (cm)		ΔWC (cm)	
	*b*	*p*	*b*	*p*		*b*	*p*	*b*	*p*
(a) Model 1									
MIP (cmH_2_O)	−4.39	0.38	0.49	0.71		0.84	0.06	0.54	0.13
MIP (%predicted)	−3.15	0.56	0.66	0.65		0.76	0.10	0.74	0.08
MEP (cmH_2_O)	−2.57	0.67	0.16	0.92		−0.15	0.78	0.24	0.58
MEP (%predicted)	0.30	0.96	−1.25	0.44		−0.03	0.96	0.52	0.23
FVC (%predicted)	0.26	0.91	−0.47	0.47		−0.11	0.60	0.11	0.51
FEV_1_ (%predicted)	−1.89	0.42	−0.47	0.46		0.20	0.32	−0.27	0.12
FEV_1_/FVC (%)	−1.73	0.19	−0.02	0.95		0.23	0.06	−0.30	**0.002**
FEF_25–75%_ (%predicted)	−9.76	**0.04**	−0.52	0.67		0.38	0.33	−0.92	**0.006**
FEF_25–75%_/FVC (%)	−11.17	**0.02**	−0.04	0.98		0.42	0.28	−0.99	**0.003**
PEF (%predicted)	−2.97	0.64	−1.30	0.44		0.39	0.48	−0.71	0.12
6MWT distance (m)	20.33	0.22	−7.74	0.08		0.20	0.89	0.15	0.90
6-MWT z-score	-	-	−0.13	0.07		0.001	0.95	0.01	0.79

(b) Model 2									
	Sex: M		ΔBMI (kg/m^2^)			ΔCC (cm)		Δ(WC/Ht)	
MIP (cmH_2_O)	−4.69	0.35	0.59		0.66	0.86	0.06	73.55	0.15
MIP (%predicted)	−3.65	0.50	0.81		0.58	0.84	0.08	90.96	0.11
MEP (cmH_2_O)	−2.24	0.71	0.10		0.95	−0.15	0.77	57.30	0.35
MEP (%predicted)	0.27	0.96	−1.23		0.44	0.01	0.99	84.63	0.17
FVC (%predicted)	0.15	0.95	−0.44		0.49	−0.10	0.64	12.95	0.59
FEV_1_ (%predicted)	−1.70	0.47	−0.53		0.40	0.18	0.38	−34.05	0.16
FEV_1_/FVC (%)	−1.44	0.29	−0.11		0.77	0.20	0.09	−33.71	**0.02**
FEF_25–75%_ (%predicted)	−8.84	**0.04**	−0.77		0.54	0.28	0.49	−102.9	**0.03**
FEF_25–75%_/FVC (%)	−10.01	**0.03**	−0.34		0.78	0.30	0.45	−102.7	**0.03**
PEF (%predicted)	−1.52	0.81	−1.68		0.33	0.27	0.63	−39.92	0.54
6MWT distance (m)	19.05	0.25	−7.43		0.09	0.29	0.84	−44.86	0.79
6-MWT z-score	-	-	−0.13		0.07	0.002	0.92	0.27	0.92

A *p* < 0.05 indicates statistical significance. The bold means a *p* < 0.05.

*Δ(WC/Ht)* absolute change in waist circumference-to-height ratio, *ΔBMI* absolute change in body mass index, *ΔCC* absolute change in chest circumference, *6MWT distance* 6-min walk test distance, *b* correlation coefficient, *FEF*_*25–75%*_ forced expiratory flow rate within 25–75% of vital capacity, *FEV*_*1*_ forced expiratory volume in 1 s, *FVC* forced vital capacity, *M* male, *m* meters, *MEP* maximal expiratory pressure, *MIP* maximal inspiratory pressure, *PEF* peak expiratory flow rate.

Model 1 included gender, ΔBMI, ΔCC, and ΔWC as independent variables.

Model 2 included gender, ΔBMI, ΔCC, andΔ(WC/Ht) as independent variables.

In Model 1, ΔWC showed a negative correlation with change in FEV_1_/FVC [Δ(FEV_1_/FVC)] (*b* = −0.3; *p* = 0.002), FEF_25–75%_ (ΔFEF_25–75%_) (*b* = −0.92; *p* = 0.006), and FEF_25–75%_/FVC [Δ(FEF_25–75%_/FVC)] (*b* = −0.99; *p* = 0.003). Male sex was shown to significantly influence ΔFEF_25–75%_ (*b* = −9.76; *p* = 0.04) and Δ(FEF_25–75%_/FVC) (*b* = −11.17; *p* = 0.02) (Table 3a).

In Model 2, Δ(WC/Ht) showed a negative correlation with Δ(FEV_1_/FVC) (*b* = −33.71; *p* = 0.02), ΔFEF_25–75%_ (*b* = −102.9; *p* = 0.04), and Δ(FEF_25–75%_/FVC) (*b* = −102.7; *p* = 0.03). Male sex was found to significantly affect ΔFEF_25–75%_ (*b* = −8.84; *p* = 0.04) and Δ(FEF_25–75%_/FVC) (*b* = −10.04; *p* = 0.03) (Table 3b).

## Discussion

The present study found that the overall BMI z-score and WC/Ht among obese children and adolescents were significantly reduced at the 1-year follow-up; however, only minor changes were observed. These minor changes in obesity indices are within normal expectations, as achieving weight control through lifestyle modifications or dietary changes—without a specific weight loss program—can be quite challenging for obese individuals. The individual differences in adherence to recommendations, as well as variations in physical activity, dietary changes, and lifestyle modifications, can also introduce confounding effects on the study results.

In terms of lung function, we observed significant increases in both the absolute and %predicted values of RMS, along with a reduction in the FEV_1_/FVC and FEF_25–75_/FVC at follow-up. A possible explanation for the increased RMS could be the heightened workload on respiratory muscles due to the excess weight carried by obese individuals. This heightened workload may elicit an adaptive response, prompting the respiratory muscles to strengthen as a compensatory mechanism to meet the increased oxygen demand.^18–20^ The present study indicated a decrease in the BMI z-score but the reduction was minimal and BMI z-score remained within the obesity range. Consequently, this compensatory mechanism may continue and contribute to the increased RMS. Despite the improvements in BMI z-score and WC/Ht, the FEV_1_/FVC and FEF_25–75_/FVC values were found to be reduced at follow-up. This decline may be explained by ongoing dysanapsis process, which refers to the disproportionate overgrowth of lung tissue relative to airway development in obese children and adolescents^21^ due to persistent obesity.

Our study also revealed differences in the absolute value of the 6-MWT distance between baseline and follow-up. The increase in absolute value can be attributed to somatic growth, particularly an increase in height, as the 6-MWT distance is significantly correlated with height.^22^ However, the z-score value for the 6-MWT distance, which is adjusted for height, did not show a significant difference. This lack of change in the z-score value may be caused by the marginal changes in BMI z-score and WC/Ht at follow-up, which likely had no impact on the z-score for the 6-MWT distance.

Alterations in lung function may result in shortness of breath or exercise intolerance.^2,3^ In our study, although notable changes in lung function were observed at the follow-up assessment, these changes were not considered clinically significant. This lack of clinical impacts may be attributed to the minor changes in both lung function and obesity indices, as well as the fact that lung function remained within normal ranges at the beginning of the study and at the 1-year follow-up.

The present study provides important data specific to the longitudinal effects of a change in obesity on lung function in obese children and adolescents. We found a change in WC or WC/Ht, which indicates abdominal adiposity, to be inversely correlated with a change in FEV_1_/FVC, FEF_25–75%_ /FVC, and FEF_25–75%_ in children and adolescents with obesity after 1 year of follow-up. In contrast, a change in BMI did not significantly affect any spirometry parameters, RMS, or 6-MWT distance.

Our results indicate that abdominal fat deposition influences lung function change more profoundly than total adiposity, as reflected by BMI. Consistent with the findings of our study, a recent large longitudinal study in middle-aged Asian population by Park, et al.^23^ reported increased WC/Ht to be significantly associated with long-term impairment of lung function. A long-term study in children and adolescents with obesity found that a change in WC had more effect on lung function than change in BMI.^7^ Many previous cross-sectional studies in children^24,25^ and adults^26–28^ also reported abdominal adiposity to be a predictor of altered lung function. The main mechanism of abdominal obesity on altered lung function is excessive fat deposition in the diaphragm and abdominal visceral organs, which exerts an adverse mechanical effect on diaphragmatic movement and lung expansion.^29,30^ In our study, a change in BMI after 1 year follow-up was not significantly related to lung function change. The reason might be that BMI indicates overall fat and non-fat components of the body, but BMI does not reflect specific body fat distribution like WC or WC/Ht. Our results seem to strongly suggest WC and WC/Ht as important markers for lung function change in obesity. Accordingly, these two obesity parameters should be measured and monitored (in addition to BW and BMI) in routine clinical practice for obese individuals. Ideally, distribution of body fat and muscle mass as measured by direct methods, such as dual-energy X-ray absorptiometry or bioelectrical impedance analysis, should be used for monitoring obese individuals. However, these measurement modalities are expensive and often not available, which makes their use often impractical in routine clinical practice.

The present study included FEF_25–75%_ /FVC, which was rarely investigated in previous studies, and found that change in WC and WC/Ht had negative association with change in FEV_1_/FVC, FEF_25–75%_ /FVC, and FEF_25–75%_, but no association with FEV_1_ or FVC.

FEV_1_/FVC and FEF_25–75%_/FVC are surrogate markers for dysanapsis, which is defined as disproportionate scaling of airway dimensions to lung volume, which in turn leads to low FEV_1_/FVC and FEF_25–75%_/FVC.^21,31^ Our 1-year follow-up study supports dysanapsis growth, which can occur in obese children and adolescents.^21^ Most studies that found negative association between FEF_25–75%_/FVC^32^ or FEV_1_/FVC^4,5,25,32^ and obesity indices had a cross-sectional design. The longitudinal Dutch PIAMA study^8^ and Swedish BAMSE cohort study^9^ also found persistent high BMI or obesity in children to be associated with lower FEV_1_/FVC, but neither of those studies evaluated FEF_25–75%_/FVC.

We also found that change in WC and WC/Ht significantly inversely affects FEF_25–75%_, which reflects small airway function. This supports the findings of recent meta-analyses that found negative association between FEF_25–75%_ and obesity status in obese children and adolescents.^4,5^ The greater effect of both WC and WC/Ht on FEF_25–75%_/FVC compared to their effect on FEV_1_/FVC, as indicated by a greater b-value for FEF_25–75%_/FVC, may reflect that obesity has more focused adverse effect on small airways. Lung function measurement that is more sensitive than spirometry for detecting impairment of small airways, such as oscillation technique, should be used to evaluate and clarify respiratory function change in obese individuals. In contrast to these findings, a longitudinal study in obese children and adolescents by van de Griendt, et al.^7^ reported no effect of change in obesity on any spirometric parameters. The actual long-term effects of fat deposition on the respiratory system and the pathophysiology of respiratory symptoms in obesity are still being investigated and debated. Further study is, therefore, needed to improve our understanding of this issue so that we can develop and improve prevention and treatment strategies.

The present study also found sex difference in small airway growth. Boys demonstrated more negative effect on FEF_25–75%_ and FEF_25–75%_/FVC. This result is consistent with those reported from previous studies that demonstrated airway growth of boys to be slower than that observed in girls.^21,33,34^

### Strengths and limitations

The strength of our study is its longitudinal design, which facilitated observation of change in the same patients over time. This is in contrast to a cross-sectional design which observes different patients at a single time point. Another strength is that we included WC and WC/Ht as obesity status variables, and FEF_25–75%_/FVC, RMS, and 6-MWT as lung function variables.

This study also has some mentionable limitations. First, data specific to other variables, such as physical activity, diet, and other environmental exposures, were not collected. Second, subgroup analysis of sex and pubertal stage was not be performed because the small size of many subgroups would need yield the statistical power needed to provide reliable statistical results. Future multicenter study in a much larger patient population is needed to confirm and expand upon the results of this study.

## Conclusions

The present longitudinal study in children and adolescents with obesity demonstrated that change in abdominal adiposity, as determined by WC and WC/Ht, significantly influences lung function change in FEV_1_/FVC, FEF_25–75%_ /FVC, and FEF_25–75%_ after 1 year of follow-up. Our results indicate that change in WC and WC/Ht adversely impacts airflow, and with likely more pronounced effect on small airways. These results permit us to suggest using WC and/or WC/Ht in addition to BW and/or BMI for monitoring obesity. Fat loss programs, especially those focused on reducing abdominal adiposity should be encouraged to prevent late lung function impairment.

## Data Availability

The datasets generated during and/or analyzed during the current study are available from the corresponding author on reasonable request and with approval of the IRB.
